# Supplementary material for: A Critical View of the Application of the APEX Software (Aqueous Photochemistry of Environmentally-Occurring Xenobiotics) to Predict Photoreaction Kinetics in Surface Freshwaters
Source: Molecules. 2019 Dec 18;25(1):9. doi: 10.3390/molecules25010009 (PMC7017383; doi:10.3390/molecules25010009)
Supplement: Supplementary file 1 [file molecules-25-00009-s001.zip › 3.2.4_gcc-4.4.0.zip!/3.2.4_gcc-4.4.0/3.2.4_gcc-4.4.0/doc/octave/html/interpreter/Specialized-Solvers.html]

Specialized Solvers - Untitled


Previous: Functions of a Matrix,
Up: Linear Algebra


---

### 18.5 Specialized Solvers

— Function File: **bicgstab** (A, b)  
— Function File: **bicgstab** (A, b, tol, maxit, M1, M2, x0)  
> This procedure attempts to solve a system of linear equations A\*x = b for x.
> The A must be square, symmetric and positive definite real matrix N\*N.
> The b must be a one column vector with a length of N.
> The tol specifies the tolerance of the method, the default value is 1e-6.
> The maxit specifies the maximum number of iterations, the default value is min(20,N).
> The M1 specifies a preconditioner, can also be a function handler which returns M\X.
> The M2 combined with M1 defines preconditioner as preconditioner=M1\*M2.
> The x0 is the initial guess, the default value is zeros(N,1).
>
> The value x is a computed result of this procedure.
> The value flag can be 0 when we reach tolerance in maxit iterations, 1 when
> we don't reach tolerance in maxit iterations and 3 when the procedure stagnates.
> The value relres is a relative residual - norm(b-A\*x)/norm(b).
> The value iter is an iteration number in which x was computed.
> The value resvec is a vector of relres for each iteration.

— Function File: **cgs** (A, b)  
— Function File: **cgs** (A, b, tol, maxit, M1, M2, x0)  
> This procedure attempts to solve a system of linear equations A\*x = b for x.
> The A must be square, symmetric and positive definite real matrix N\*N.
> The b must be a one column vector with a length of N.
> The tol specifies the tolerance of the method, default value is 1e-6.
> The maxit specifies the maximum number of iteration, default value is MIN(20,N).
> The M1 specifies a preconditioner, can also be a function handler which returns M\X.
> The M2 combined with M1 defines preconditioner as preconditioner=M1\*M2.
> The x0 is initial guess, default value is zeros(N,1).
